# Supplementary material for: Aurora A regulates the material property of spindle poles to orchestrate nuclear organization at mitotic exit
Source: EMBO J. 2025 Sep 12;44(23):6797–831. doi: 10.1038/s44318-025-00564-4 (PMC12669695; doi:10.1038/s44318-025-00564-4)
Supplement: Supplementary file 1 — Appendix [file 44318_2025_564_MOESM1_ESM.pdf]

## Appendix data for:

# *Aurora A regulates the material property of spindle poles to orchestrate nuclear organization at mitotic exit*

## Table of contents


| | Page |
|---|---|
| **Appendix Figure S1: The occurrence of misshapen nuclei in Aurora A-inhibited cells is not due to weak NuMA levels in the nucleus** | **1-2** |
| **Appendix Figure S2: Acute Aurora A inactivation does not enrich the levels of key PCM matrix- proteins** | **3** |
| **Appendix Figure S3: Aurora A controls organization of NuMA at the poles** | **4** |
| **Appendix Figure S4: Asymmetry in NuMA accumulation at the poles correlates to asymmetric behavior to NuMA material properties in Plk4 inhibited cells** | **5-6** |
| **Appendix Figure S5: Reorganization of chromosome arm contacts upon Aurora A-inhibition** | **7-8** |
| **Appendix Table S1: List of plasmids used in this study** | **9** |
| **Appendix Table S2: List of primers used in this study** | **10-12** |
| **Appendix Reference:** | **13** |

# Fig. S1

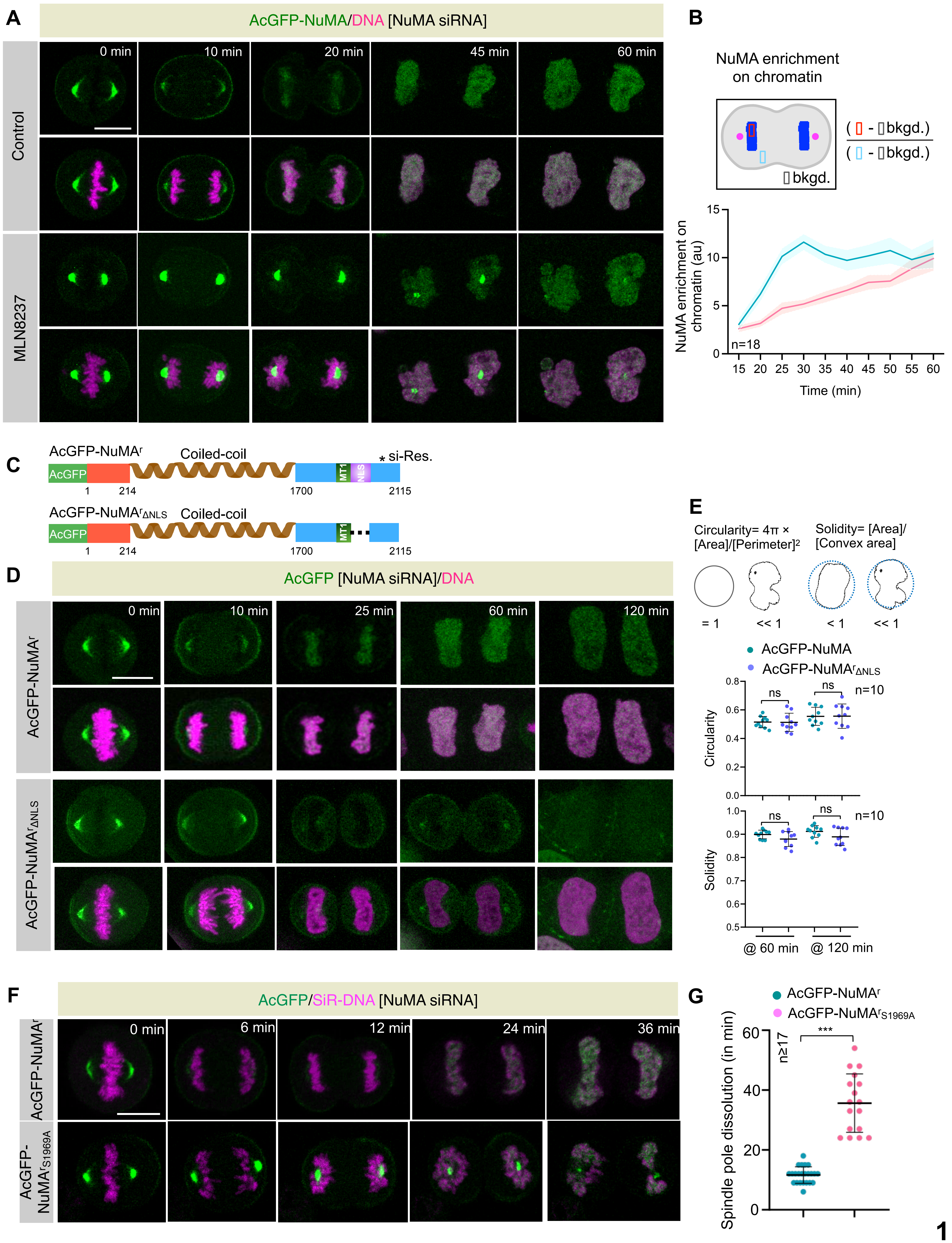

A
AcGFP-NuMA/DNA [NuMA siRNA]
0 min
10 min
20 min
45 min
60 min
Control
MLN8237
B
NuMA enrichment on chromatin
bkgd.
NuMA enrichment on chromatin (au)
n=18
Time (min)
C
AcGFP-NuMAr
Coiled-coil
si-Res.
AcGFP
MT1
NLS
1
214
1700
2115
AcGFP-NuMArΔNLS
D
AcGFP [NuMA siRNA]/DNA
0 min
10 min
25 min
60 min
120 min
AcGFP-NuMAr
AcGFP-NuMArΔNLS
E
Circularity= 4π × [Area]/[Perimeter]2
Solidity= [Area]/[Convex area]
= 1
<< 1
< 1
<< 1
AcGFP-NuMA
AcGFP-NuMArΔNLS
ns
n=10
Circularity
Solidity
@ 60 min
@ 120 min
F
AcGFP/SiR-DNA [NuMA siRNA]
0 min
6 min
12 min
24 min
36 min
AcGFP-NuMAr
AcGFP-NuMArS1969A
G
AcGFP-NuMAr
AcGFP-NuMArS1969A
Spindle pole dissolution (in min)
n≥17
***

**Appendix Figure S1**

**The occurrence of misshapen nuclei in Aurora A-inhibited cells is not due to weak NuMA levels in the nucleus**

(A) Representative images from the confocal live-cell imaging of HeLa cells stably coexpressing siRNA-resistant NuMA with AcGFP [AcGFP-NuMA$^{r}$; (green)] and mCherry-H2B (magenta), and are transfected with NuMA siRNA for 72 h to deplete the endogenous protein. These cells are either left untreated or acutely treated with MLN8237 for 45 min before live-cell imaging analysis (A).

(B) Schematic of the method to quantify nuclear AcGFP-NuMA levels over time in untreated versus MLN8237-treated cells, and the outcome of such analysis. Curves and shaded areas indicate mean ± SEM. The p-values for between control and MLN8237-treated cells from 25-50 min post metaphase-to-anaphase transition is <0.001, and thus significant. However, the p-values at 55 min and 60 min is 0.5355, 0.8214, and therefore non-significant (ns).

(C) Domain organization of AcGFP-tagged and siRNA-resistant wild-type full-length NuMA (AcGFP-NuMA$^{r}$), and NuMA lacking nuclear localization signal (AcGFP-NuMA$^{r}_{\Delta NLS}$).

(D) Representative images from the confocal live-cell imaging of HeLa cells transiently transfected with plasmids expressing siRNA-resistant AcGFP-NuMA$^{r}$ (green) or AcGFP-NuMA$^{r}_{\Delta NLS}$ (green) with mCherry-H2B (magenta) and are transfected with NuMA siRNA for 72 h to deplete the endogenous protein.

(E) Nuclear shape analysis [circularity and solidity] from the confocal live-cell imaging of cells, as mentioned in panel D @ 60 min or 120 min post metaphase-to-anaphase transition, as indicated. Error bars: mean ± SD. Exact p-values from left to right for circularity are p=0.9424 (ns), p=0.9626 (ns), and solidity are p=0.1155 (ns), p=0.1211 (ns).

(F, G) Representative images from the confocal live-cell imaging of HeLa cells stably coexpressing either AcGFP-NuMA$^{r}$ or AcGFP-NuMA$^{r}_{S1969A}$ (green) and probed for SiR-DNA (magenta) to visualize chromosome ensemble during anaphase. Recording was started 72 h post-transfection for control siRNA and NuMA siRNA (F). The graph on the right represents the quantification of AcGFP-NuMA or AcGFP-NuMA$^{r}_{S1969A}$ dissolution time (in min) at the poles with respect to (w.r.t.) metaphase-to-anaphase transition (G). Error bars: mean ± SD. Exact p-value is ***p<0.001.
p-values are denoted as follows: ns- p>0.05; ***-p<0.001 as determined by two-tailed unpaired Student's t-test. Scale bars in (A, D, F) represent 10 $\mu$m.

Fig. S2

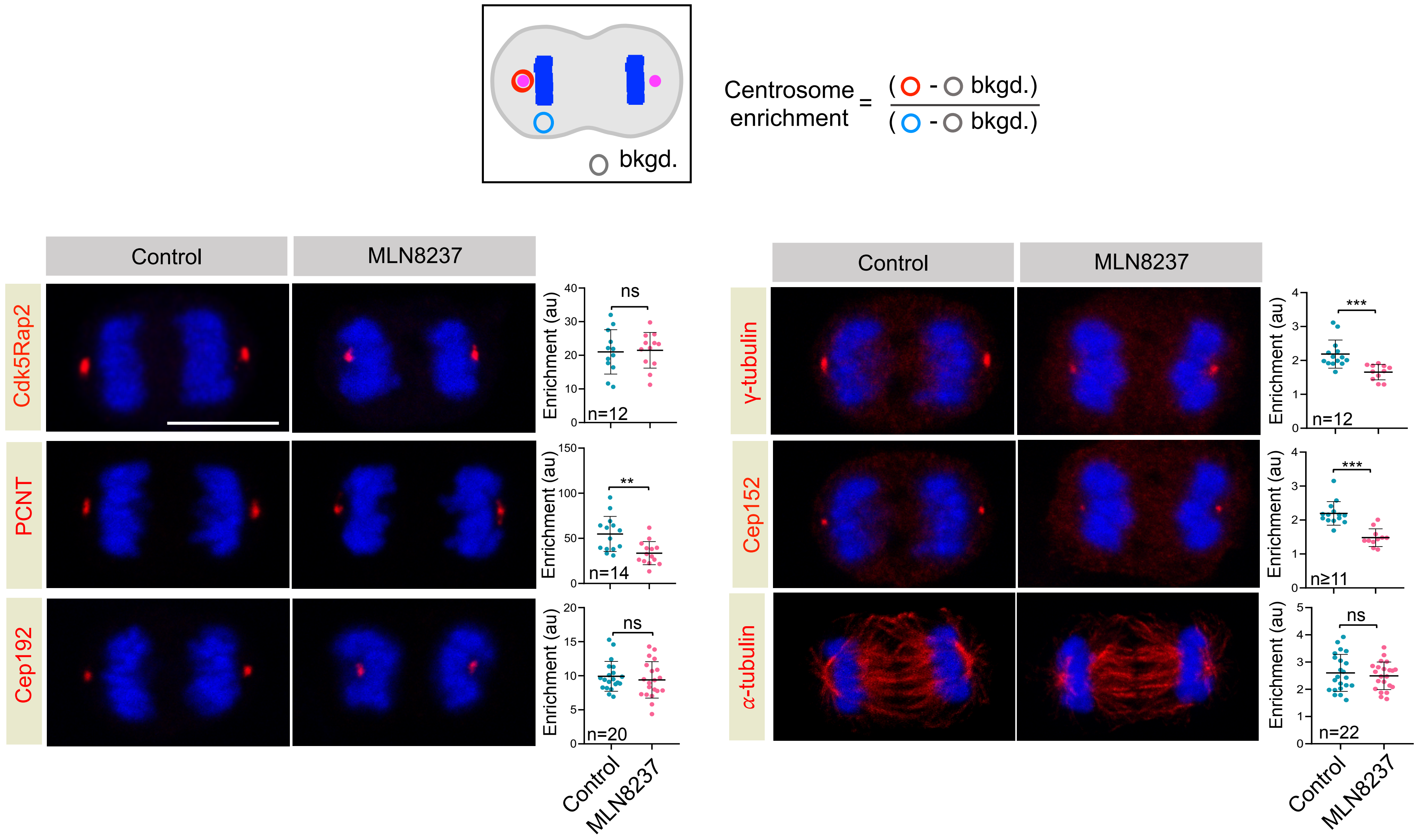


**Appendix Figure S2**

**Acute Aurora A inactivation does not enrich the levels of key PCM matrix- proteins**

Schematic representation of the method, and the quantification of spindle pole intensity of various centrosomal components (in au) stained for IF microscopy analysis. Cells were stained with anti-Cdk5Rap2, anti-PCNT, anti-Cep192, anti-$\gamma$-tubulin, anti-Cep152, and anti-$\alpha$-tubulin antibodies (all in red). DNA is shown in blue. The quantification is shown on the right; bars indicate mean ± SD. Exact p-values are p=0.8443 (ns) for Cdk5Rap2, **p=0.0015 for PCNT, p=0.5099 (ns) for Cep192, ***p<0.001 for $\gamma$-tubulin, ***p<0.001 for Cep152, p=0.6655 (ns) for $\alpha$-tubulin.

p-values are denoted as follows: ns- p>0.05; **-p<0.01; ***-p<0.001 as determined by two-tailed unpaired Student's t-test. Scale bar represents 10 $\mu$m.

Fig. S3

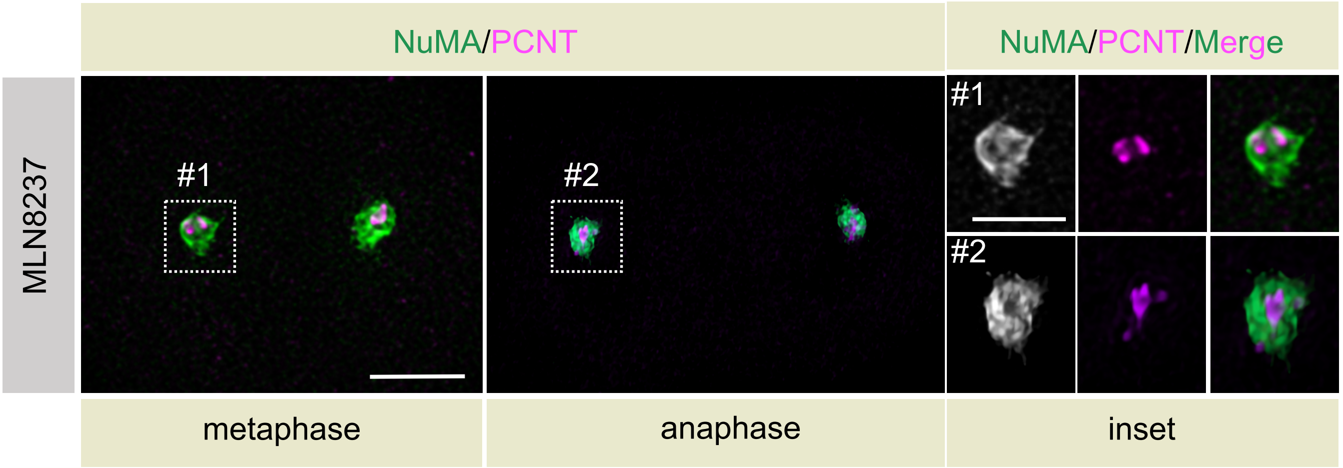


**Appendix Figure S3**

<u>**Aurora A controls organization of NuMA at the poles**</u>

Representative images from the super-resolution 3D-SIM$^2$ analysis of HeLa cells immunostained with anti-NuMA (green) and anti-PCNT (magenta) during metaphase and anaphase upon acute treatment with MLN8237. Insets on the right show the pole localized NuMA (grey) and PCNT (magenta). Related to Fig 3G. n≥10 cells were analysed for MLN8237 in metaphase and anaphase, and the representative image is shown above.

Scale bars represent 5 $\mu$m for the cell and 2 $\mu$m for the insets.

# Fig. S4

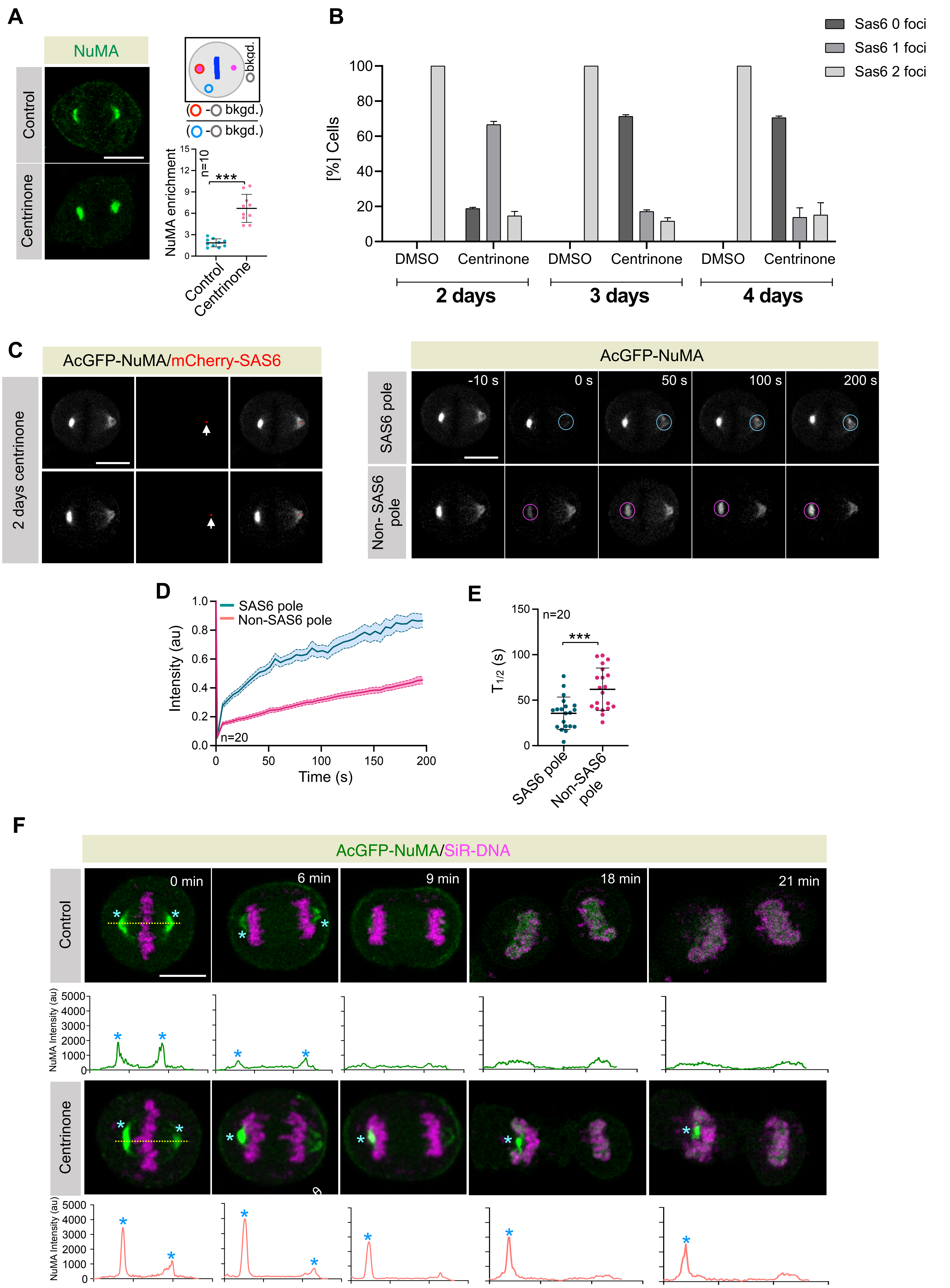

A
NuMA
Control
Centrinone
bkgd.
( -  bkgd.)
( -  bkgd.)
NuMA enrichment
n=10
***
B
Sas6 0 foci
Sas6 1 foci
Sas6 2 foci
[%] Cells
DMSO
Centrinone
2 days
3 days
4 days
C
AcGFP-NuMA/mCherry-SAS6
2 days centrinone
AcGFP-NuMA
-10 s
0 s
50 s
100 s
200 s
SAS6 pole
Non- SAS6 pole
D
SAS6 pole
Non-SAS6 pole
Intensity (au)
Time (s)
n=20
E
n=20
***
T1/2 (s)
SAS6 pole
Non-SAS6 pole
F
AcGFP-NuMA/SiR-DNA
0 min
6 min
9 min
18 min
21 min
Control
Centrinone
NuMA Intensity (au)

**Appendix Figure S4**

**Asymmetry in NuMA accumulation at the poles correlates to asymmetric behavior to NuMA material properties in Plk4 inhibited cells**

(A) Immunofluorescence (IF) analysis of control and centrinone (Plk4 inhibitor)-treated HeLa cells. These cells were stained with anti-NuMA antibody after 4 days of 100 nM centrinone treatment, and the pole accumulation of NuMA is determined as shown in schematic. Note a robust NuMA accumulation at the poles in cells that were incubated with centrinone, analogous to when cells are inhibited for Aurora A kinase. Error bars: mean ± SD. Exact p-value is ***p<0.001.

(B) Quantification of the fraction of cells with 0, 1, or 2 centrosomes, assessed by the IF analysis with centriole-specific antibody anti-SAS6, after incubation of cells either with control (DMSO) or centrinone for 2-4 days, as indicated. Error bars: mean ± SD from two independent experiments (n>500 cells each).

(C) Representative images of HeLa cells coexpressing AcGFP-NuMA$^r$ and mCherry-SAS6, which are treated with centrinone for 2 days to achieve centrosome asymmetry during mitosis; shown by mCherry-SAS6 signal-indicated by arrow. On the right, these cells are used for FRAP analysis of the AcGFP-NuMA signal at the poles. Note the faster recovery of AcGFP-NuMA at the pole with SAS6 signal (blue circle), in comparison to the pole without SAS6 signal (magenta circle).

(D) The AcGFP recovery profile of the bleached area plotted for 200 s for AcGFP-NuMA$^r$ signal at the pole with SAS6 signal (blue circle), or at the pole with no SAS6 signal (magenta circle). Curves and shaded areas indicate mean ± SEM.

(E) The half-time of recovery [$T_{1/2}$] in of AcGFP-NuMA$^r$ at poles with or without SAS6, as indicated. Error bars: mean ± SD. Exact p-value is ***p<0.001.

(F) Confocal live-cell imaging of HeLa cells stably coexpressing AcGFP-NuMA (green) and probed with SiR DNA (magenta) that were treated with centrinone for 2 days. Timepoint 0 indicates metaphase-to-anaphase transition. In contrast to control cells, centrinone-treated cells show abnormal accumulation of NuMA at one pole during anaphase. The asymmetric pole accumulation of NuMA for one representative cell is shown using line-scan analysis. Please note that these cells were utilized to calculate the chromosome ensemble bending angle in Figure 7H.
p-values are denoted as ***-p<0.001 as determined by two-tailed unpaired Student's t-test. Scale bars in (A, C, F) represent 10 $\mu$m.

Fig. S5

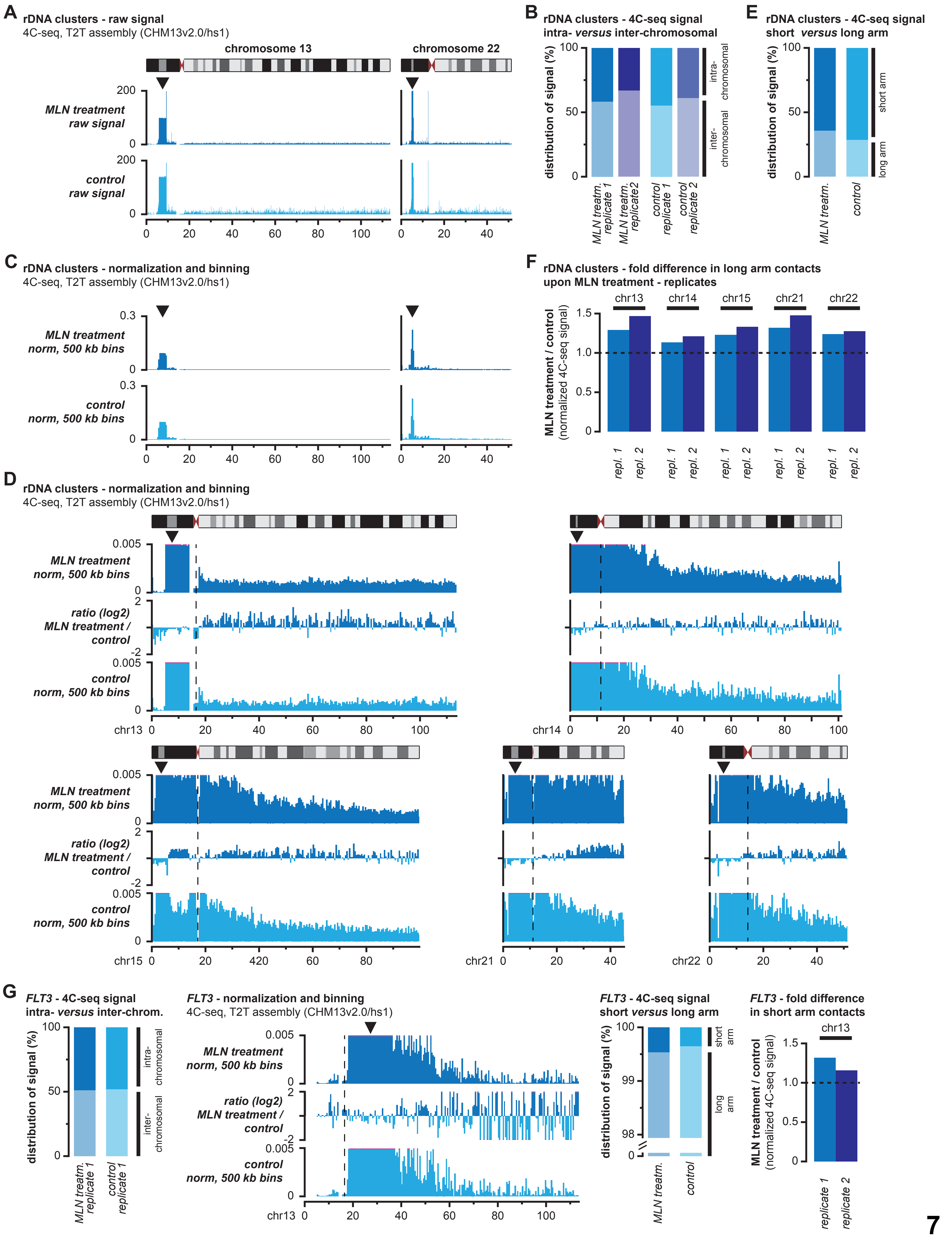

A
rDNA clusters - raw signal
4C-seq, T2T assembly (CHM13v2.0/hs1)
chromosome 13
chromosome 22
MLN treatment raw signal
control raw signal
B
rDNA clusters - 4C-seq signal
intra- versus inter-chromosomal
distribution of signal (%)
intra-chromosomal
inter-chromosomal
MLN treatm. replicate 1
MLN treatm. replicate2
control replicate 1
control replicate 2
E
rDNA clusters - 4C-seq signal
short versus long arm
short arm
long arm
MLN treatm.
control
C
rDNA clusters - normalization and binning
4C-seq, T2T assembly (CHM13v2.0/hs1)
MLN treatment norm, 500 kb bins
control norm, 500 kb bins
F
rDNA clusters - fold difference in long arm contacts upon MLN treatment - replicates
chr13
chr14
chr15
chr21
chr22
MLN treatment / control (normalized 4C-seq signal)
repl. 1
repl. 2
D
rDNA clusters - normalization and binning
4C-seq, T2T assembly (CHM13v2.0/hs1)
MLN treatment norm, 500 kb bins
ratio (log2) MLN treatment / control
control norm, 500 kb bins
chr13
chr14
chr15
chr21
chr22
G
FLT3 - 4C-seq signal
intra- versus inter-chrom.
distribution of signal (%)
intra-chromosomal
inter-chromosomal
MLN treatm. replicate 1
control replicate 1
FLT3 - normalization and binning
4C-seq, T2T assembly (CHM13v2.0/hs1)
MLN treatment norm, 500 kb bins
ratio (log2) MLN treatment / control
control norm, 500 kb bins
chr13
FLT3 - 4C-seq signal
short versus long arm
short arm
long arm
MLN treatm.
control
FLT3 - fold difference in short arm contacts
chr13
MLN treatment / control (normalized 4C-seq signal)
replicate 1
replicate 2

**Appendix Figure S5**

**Reorganization of chromosome arm contacts upon Aurora A-inhibition**

(A) Examples of raw intra-chromosomal 4C-seq signal for the rDNA clusters on the acrocentric chromosomes 13 and 22 in MLN8237-treated cells and controls at 1.5 h after nocodazole release. Chromosome ideograms are depicted above, with the burgundy arrowheads indicating the position of the centromere and the black arrowheads indicating the centre of the rDNA cluster on the short arm where the 4C-seq viewpoints are located.

(B) Quantification of the combined 4C-seq signal on the five acrocentric chromosomes (“intra-chromosomal”) versus the other chromosomes (“inter-chromosomal”) for replicate samples at 1.5 h after nocodazole release.

(C) Examples of normalized and binned 4C-seq signal at 500 kb resolution for the rDNA clusters on chromosomes 13 and 22. Normalization was performed for each chromosome individually. Further annotation as in panel A.

(D) Zoomed-in normalized and binned intra-chromosomal 4C-seq signal at 500 kb resolution for the rDNA clusters on the five acrocentric chromosomes. The difference between panels is indicated in-between (log2 ratio). Normalization was performed for each chromosome individually. Further annotation as in panel A.

(E) Quantification of the combined 4C-seq signal on the short arms of the five accrocentric chromosomes, where the rDNA clusters are located (i.e., representing intra-arm contacts), versus the long arms (i.e., representing inter-arm contacts) in MLN8237-treated cells and controls at 1.5 h after nocodazole release.

(F) Quantification of differences in normalized long arm contacts for replicate samples at 1.5 h after nocodazole release.

(G) 4C-seq analysis of a viewpoint in the promoter of the *FLT3* gene, located close to the centromere on the long arm of chromosome 13. Outer left: quantification of 4C-seq signal on chromosome 13 (“intra-chromosomal”) versus the other chromosomes (“inter-chromosomal”) at 1.5 h after nocodazole release (see panel B). Inner left: zoomed-in normalized, and binned intra-chromosomal 4C-seq signal at 500 kb resolution (see panel D). Inner right: quantification of 4C-seq signal on the long arm, where *FLT3* is located (i.e., representing intra-arm contacts), versus the short arm (i.e., representing inter-arm contacts) in MLN8237-treated cells and controls at 1.5 h after nocodazole release (see panel E). Outer right: quantification of differences in normalized short arm contacts for replicate samples at 1.5 h after nocodazole release.

**Appendix Table S1. List of plasmids used in this study**

| Name of the plasmid | Reference or source | Identifier |
|---|---|---|
| AcGFP-NuMA$^{r}$ | This study | |
| AcGFP-NuMA$^{r}$ $_{(1-1699)}$ | This study | |
| AcGFP-NuMA$^{r}$ $_{(1700-2115)}$ | This study | |
| AcGFP-NuMA$^{r}$ $_{(Aro>A)}$ | This study | |
| AcGFP-NuMA$^{r}$ $_{(R>G)}$ | This study | |
| AcGFP-NuMA$^{r}$ $_{(S1969A)}$ | This study | |
| AcGFP-NuMA$^{r}$ $_{NC}$ | This study | |
| AcGFP-NuMA$^{r}$ $_{\Delta DBD}$ | This study | |
| AcGFP-NuMA$^{r}$ $_{\Delta NLS}$ | This study | |
| H2B-mCherry | Addgene | Plasmid# 21044 |
| HNRNPA1c-mCherry-SspB | This study | Subcloned into the IRES vector from Addgene Plasmid# 122668 |
| HNRNPA1c-mCherry-SspB | Bracha et al., 2018 | |
| iLid-GFP-FTH1 | Bracha et al., 2018 | |
| Kaede-NuMA | This study | |
| mEos-NuMA | This study | |
| mEos-NuMA $_{(S1969A)}$ | This study | |
| NuMA$_{C\text{-}ter(Aro>A)}$-mCherry-SspB | This study | |
| NuMA$_{C\text{-}ter(Q>G)}$-mCherry-SspB | This study | |
| NuMA$_{C\text{-}ter(R>G)}$-mCherry-SspB | This study | |
| NuMA$_{C\text{-}ter}$-mCherry-SspB | This study | |
| NuMA$_{(1-2057)}$-mCherry-SspB | This study | |
| SNAP-NuMA | This study | |

## Appendix Table S2. List of primers used in this study

| Name of the primer | Source | Sequence (5'→3') |
|---|---|---|
| AgeI Fwd NuMA | Eurofins | GCACCGGTATGACACTCCACGCCACCCGGGGG |
| NotI Rev NuMA 2115 | Eurofins | GCGGCCGCTTAGTGCTTTGCCTTGCCCTTGGCTCG |
| AgeI Fwd NuMA 1700 | Eurofins | GCACCGGTTTCCAGGTGGCAACTGATGC |
| AgeI Fwd NuMA 706 | Eurofins | GCACCGGTGCCCTCAAAGAGTCCTTGAAGG |
| EcoRI Rev NuMA 705 | Eurofins | GCGAATTCCTGGAGCTGCTCCTGGAGCTGG |
| EcoRI Fwd NuMA 1700 | Eurofins | GCGAATTCTTCCAGGTGGCAACTGATGC |
| NotI Rev NuMA 1699 | Eurofins | GCGCGGCCGCTTATTTGCCCAGGTCTCGAAGCT |
| EcoRI Rev NuMA 1699 WO STOP | Eurofins | GCGAATTCTTTGCCCAGGTCTCGAAGCTGCTGG |
| EcoRI Fwd NuMA 1700 | Eurofins | GCGAATTCCCCTCTGCGGCAGAAGGTGG |
| EcoRI Fwd NuMA (R>G) 1700 | Eurofins | GCGAATTCTTTCAAGTTGCTACCGATGCCC |
| EcoRI Fwd NuMA (Q>G) 1700 | Eurofins | GCGAATTCTTCGGAGTCGCTACCGACGCACTCAAATCC |
| EcoRI Fwd NuMA (Aro>A) 1700 | Eurofins | GCGAATTCGCACAAGTCGCTACGGACGCCC |
| BamHI Rev NuMA 2115 | Eurofins | GCGGATCCTTAGTGCTTTGCCTTGCCCTTGGCTCG |
| BamHI Rev NuMA (R>G) 2115 | Eurofins | GCGGATCCTCAATGTTTGGCTTTCCCTTTAGCGCCGGG |
| BamHI Rev NuMA (Q>G) 2115 | Eurofins | GCGGATCCTCAATGTTTGGCTTTCCCTTTTGCGCGTGG |
| BamHI Rev NuMA (Aro>A) 2115 | Eurofins | GCGGATCCCTAATGTTTAGCTTTACCTTTAGCTCTCG |
| NuMA 1985 ΔNLS Rev Overlap | Eurofins | TGGTGGCGCGCTGCTGCCGG |
| NuMA 2005 ΔNLS FwD Overlap | Eurofins | GCAGCGCGCCACCAGCTGTTTCCCAC |
| NuMA Q>G S1969A FwD | Eurofins | CTTTGAGAAGAGCGGCAATGGGCCCCATTGGG |
| EcoRV Fwd Aurora A | Eurofins | GCGATATCATGAGTCACAAGCCGGTTCAGAATCAG |
| NotI Rev AcGFP | Eurofins | CGCGCGGCCGCGGATCCTCACTTG |
| AgeI Fwd mCherry | Eurofins | GCACCGGTAATGGTGTCTAAAGGCGAGGAGGATAAC |

| | | |
|---|---|---|
| NotI Rev sspB | Eurofins | GCGCGGCCGCTTAACCAATATTCAGCTCGTCATAG |
| NheI Fwd HNRNPA1c | Eurofins | GCGCTAGCATGGCTAGTGCTTCATCCAGCCAAAG |
| XhoI FwD NuMA 1700 | Eurofins | GCCTCGAGATGTTCCAGGTGGCAACTGATGCTTTAAAG AGC |
| AgeI Rev NuMA 2057 IDR | Eurofins | TACCGGTCCCTTGGGTGTGTTGAGGATGCTGAAGGCC |
| EcoR1 Fwd NuMA | Eurofins | GCGAATTCCATGACACTCCACGCCACCCGGGG |
| XhoI FwD R>G NuMA 1700 | Eurofins | GCCTCGAGATGTTTCAAGTTGCTACCGATGCCCTGAAAA GTGG |
| AgeI Rev R>G NuMA 2057 | Eurofins | TTACCGGTCCCTTTGGGGTATTCAGTATAGAGAAAGCCA TGG |
| XhoI FwD Q>G NuMA 1700 | Eurofins | GCCTCGAGATGTTCGGAGTCGCTACCGACGCACTCAAA TCC |
| AgeI Rev Q>G NuMA 2057 | Eurofins | TTACCGGTCCCTTTGGGGTATTAAGAATGGAAAATGCC |
| XhoI Fwd Aro>A NuMA 1700 | Eurofins | GCCTCGAGATGGCACAAGTCGCTACGGACGCCC |
| AgeI Rev Aro>A NuMA 2057 | Eurofins | TTACCGGTCCCTTTGGGGTATTCAAAATGGAAGC |
| Nup107 BamHI fwd | Eurofins | GCGGATCCTATGGACAGGAGTGGCTTTGGAGAG |
| Nup107 NotI rev | Eurofins | GCGCGGCCGCCTATAACTGAATTTCATACCCTAATGGG |
| CycB EcoRV fwd | Eurofins | GCGATATCATGGCGCTCCGAGTCACCAGGAACTCG |
| CycB AgeI rev | Eurofins | GCACCGGTTACCTTTTCAAGAGGTTTTGGTAG |
| AgeI fwd Aurora A67aa Fwd | Eurofins | GCACCGGTAGTCACAAGCCGGTTCAGAATCAG |
| S1969A NuMA | Eurofins | CCTGCGCCGAGCCGCCATGCAGCCAAT |
| XhoI rev Aurora A | Eurofins | CGCTCGAGAGACTGTTTGCTAGCTGATTCTTTG |
| AgeI Fwd Fib-7 | Eurofins | GCACCGGTATGAAGCCAGGATTCAGTCCCCGTGGG |
| BamHI Fib-7 | Eurofins | GCGGATCCTCAGTTCTTCACCTTGGGGGGTGGCC |
| EcoR1 fwd NuMA (for mEos) | Eurofins | GCGAATTCCATGACACTCCACGCCACCCGGGG |
| NuMA CT BamHI rev (for mEos) | Eurofins | GCGGATCCTTAGTGCTTTGCCTTGCCCTTGGCTCG |
| SNAP Kpn1 fwd | Eurofins | GCGGTACCATGGACAAAGACTGCGAAATGAAGC |

| | | |
|---|---|---|
| SNAP Not1 fwd NuMA cloning | Eurofins | GCGCGGCCGCCACCCAGCCCAGGCTTGCCCAGTCTGTG |
| siRNA res NuMA 8 Stealth rev primer | Eurofins | CAGACCGCGTGCTGTAAAAAGAAGAATTGGCGCTATCGGGCTCTTCCACATCTAGC |
| Lamin Fwd Age1 | Eurofins | GCACCGGTATGGCGACTGCGACCCCCGTGC |
| Lamin Rev Bamh1 | Eurofins | GCGGATCCCTACATAATTGCACAGCTTCTATTGG |
| PR150 mCh NuMA fwd | Eurofins | GCAGGCGGCCGCATGACACTCCACGCCACC |
| XbaI rev NuMA | Eurofins | CGTCTAGATTAGTGCTTTGCCTTGCCCTTGGC |
| EcoR1 fwd Kaede | Eurofins | CGGAATTCATGGTGAGTCTGATTAAACCAGAAATG |
| Not1 rev Kaede | Eurofins | CGGCGGCCGCCCTTGACGTTGTCCGGCAATCCAG |
| Human rDNA 4C-seq viewpoint iF | IDT | TACACGACGCTCTTCCGATCTATGCAAATGTCAGCCTGG |
| Human rDNA 4C-seq viewpoint iR | IDT | ACTGGAGTTCAGACGTGTGCTCGCTTTTGTGACTCTCTCAA |
| Human *FLT3* 4C-seq viewpoint iF | IDT | TACACGACGCTCTTCCGATCTAATTGCCTTGGTTGACATTG |
| Human FLT3 4C-seq viewpoint iR | IDT | ACTGGAGTTCAGACGTGTGCTTCTCCCAATTCTAGGAATTCC |
| 4C-seq universal step2 F | IDT | AATGATACGGCGACCACCGAGATCTACACTCTTTCCCTACACGACGCTCTTCCGATCT |
| 4C-seq index1 step2 R | IDT | CAAGCAGAAGACGGCATACGAGATCGTGATGTGACTGGAGTTCAGACGTGTGCT |
| 4C-seq index2 step2 R | IDT | CAAGCAGAAGACGGCATACGAGATACATCGGTGACTGGAGTTCAGACGTGTGCT |

**Appendix Reference**

Bracha D, Walls MT, Wei MT, Zhu L, Kurian M, Avalos JL, Toettcher JE, Brangwynne CP (2018) Mapping Local and Global Liquid Phase Behavior in Living Cells Using Photo-Oligomerizable Seeds. Cell 175: 1467-1480 e13
