# Supplementary material for: Aurora A regulates the material property of spindle poles to orchestrate nuclear organization at mitotic exit
Source: EMBO J. 2025 Sep 12;44(23):6797–831. doi: 10.1038/s44318-025-00564-4 (PMC12669695; doi:10.1038/s44318-025-00564-4)
Supplement: Supplementary file 3 — Movie EV1 [file 44318_2025_564_MOESM3_ESM.zip › Movie EV1/Movie EV1.docx]

**Movie EV1**: Confocal live-cell imaging of HeLa cells stably coexpressing AcGFP-LaminB1 (green) and mCherry-H2B (magenta), and are treated with DMSO (related to Fig. 1A). Time, t = 0, represents the metaphase to anaphase transition. Time is in h:min format; Playback 3 frames/s.
