# Supplementary material for: Aurora A regulates the material property of spindle poles to orchestrate nuclear organization at mitotic exit
Source: EMBO J. 2025 Sep 12;44(23):6797–831. doi: 10.1038/s44318-025-00564-4 (PMC12669695; doi:10.1038/s44318-025-00564-4)
Supplement: Supplementary file 4 — Movie EV2 [file 44318_2025_564_MOESM4_ESM.zip › Movie EV2/Movie EV2.docx]

**Movie EV2**: Confocal live-cell imaging of HeLa cells stably coexpressing AcGFP-LaminB1 (green) and mCherry-H2B (magenta) and were acutely treated with MLN8237 (related to Fig. 1A). Note the bending of chromatin in anaphase and distorted nuclear morphology in the G1 phase compared to the DMSO-treated condition (Movie EV1). Time, t = 0, represents the metaphase to anaphase transition. Time is in h:min format; Playback 3 frames/s. This movie corresponds to the data shown in Fig. 1A.
