# Supplementary material for: Aurora A regulates the material property of spindle poles to orchestrate nuclear organization at mitotic exit
Source: EMBO J. 2025 Sep 12;44(23):6797–831. doi: 10.1038/s44318-025-00564-4 (PMC12669695; doi:10.1038/s44318-025-00564-4)
Supplement: Supplementary file 5 — Movie EV3 [file 44318_2025_564_MOESM5_ESM.zip › Movie EV3/Movie EV3.docx]

**Movie EV3**: Three-dimensional rendered sections (related to Fig. 2A) showing nuclei (shown in yellow) and endogenous NuMA (shown in green) in the G1 phase in HeLa cells which were treated with DMSO. The DNA is stained using Hoechst 33342. Nuclear-enriched NuMA is not visible here because NuMA intensity was thresholded based on NuMA accumulation at the spindle pole at the same cell cycle stage in the MLN8237-treated condition (see Movie EV4). To create such movies in Imaris (Oxford Instruments), 10-15 z-sections with a step size of 1 µm were taken and processed.
