# Supplementary material for: Aurora A regulates the material property of spindle poles to orchestrate nuclear organization at mitotic exit
Source: EMBO J. 2025 Sep 12;44(23):6797–831. doi: 10.1038/s44318-025-00564-4 (PMC12669695; doi:10.1038/s44318-025-00564-4)
Supplement: Supplementary file 6 — Movie EV4 [file 44318_2025_564_MOESM6_ESM.zip › Movie EV4/Movie EV4.docx]

**Movie EV4**: Three-dimensional rendered sections (related to Fig. 2A) showing nuclei (shown in yellow) and endogenous NuMA (shown in green) in the G1 phase in HeLa cells that were acutely treated with MLN8237. The nucleus is stained using Hoechst 33342. Note the accumulation of NuMA at the spindle pole and the bending of the nucleus around the spindle pole enriched NuMA at the G1 phase compared to the control (Movie EV3).
