# Supplementary material for: Aurora A regulates the material property of spindle poles to orchestrate nuclear organization at mitotic exit
Source: EMBO J. 2025 Sep 12;44(23):6797–831. doi: 10.1038/s44318-025-00564-4 (PMC12669695; doi:10.1038/s44318-025-00564-4)
Supplement: Supplementary file 7 — Movie EV5 [file 44318_2025_564_MOESM7_ESM.zip › Movie EV5/Movie EV5.docx]

**Movie EV5**: Three-dimensional rendered sections (related to Fig. 6B) of cells expressing AcGFP-NuMA and nuclei (shown in yellow) at the G1 phase in HeLa Kyoto cells transiently transfected with AcGFP-NuMA. The nucleus is stained using Hoechst 33342. Nuclear-enriched NuMA is not visible here because NuMA intensity was thresholded based on Kaede-NuMA accumulation at the spindle pole (see Movie EV6) in the same cell cycle stage.
