# Supplementary material for: Aurora A regulates the material property of spindle poles to orchestrate nuclear organization at mitotic exit
Source: EMBO J. 2025 Sep 12;44(23):6797–831. doi: 10.1038/s44318-025-00564-4 (PMC12669695; doi:10.1038/s44318-025-00564-4)
Supplement: Supplementary file 8 — Movie EV6 [file 44318_2025_564_MOESM8_ESM.zip › Movie EV6/Movie EV6.docx]

**Movie EV6**: Three-dimensional rendered sections (related to Fig. 6B) showing nuclei (shown in yellow) and Kaede-NuMA localized at spindle poles (shown in green) in the G1 phase in HeLa Kyoto cells transiently transfected with Kaede-NuMA. The nucleus is stained using Hoechst 33342. Note the accumulation of Kaede-NuMA at the spindle pole and the bending of the nucleus around it in the G1 phase compared to the control (Movie EV5).
