# Supplementary material for: Aurora A regulates the material property of spindle poles to orchestrate nuclear organization at mitotic exit
Source: EMBO J. 2025 Sep 12;44(23):6797–831. doi: 10.1038/s44318-025-00564-4 (PMC12669695; doi:10.1038/s44318-025-00564-4)
Supplement: Supplementary file 9 — Source data Fig. 1 [file 44318_2025_564_MOESM9_ESM.zip › Figure 1/1F.pptx]

## Slide 1
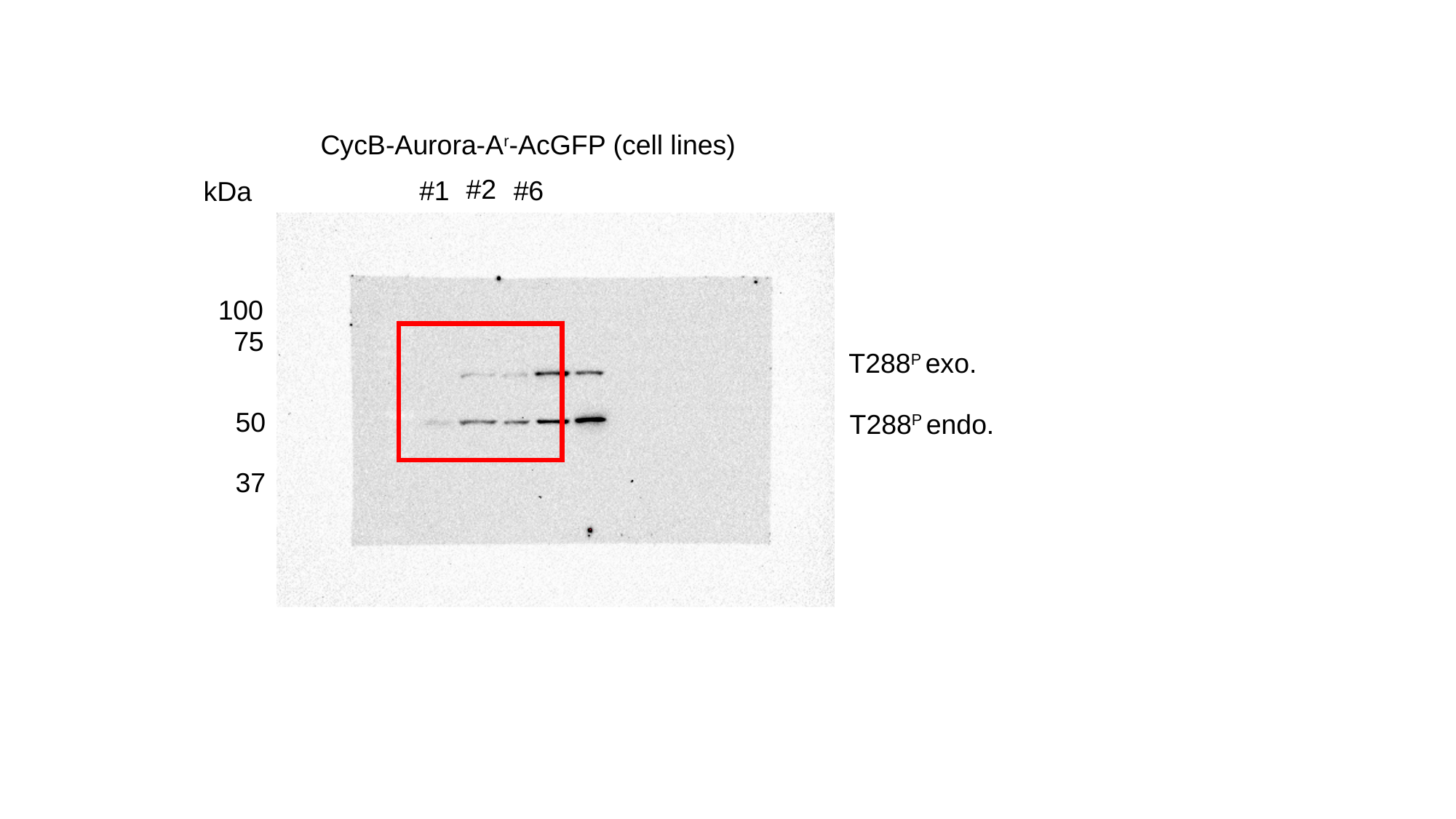

CycB-Aurora-Ar-AcGFP (cell lines)
#2
#1
#6
kDa
100
75
T288P exo.
50
T288P endo.
37

## Slide 2
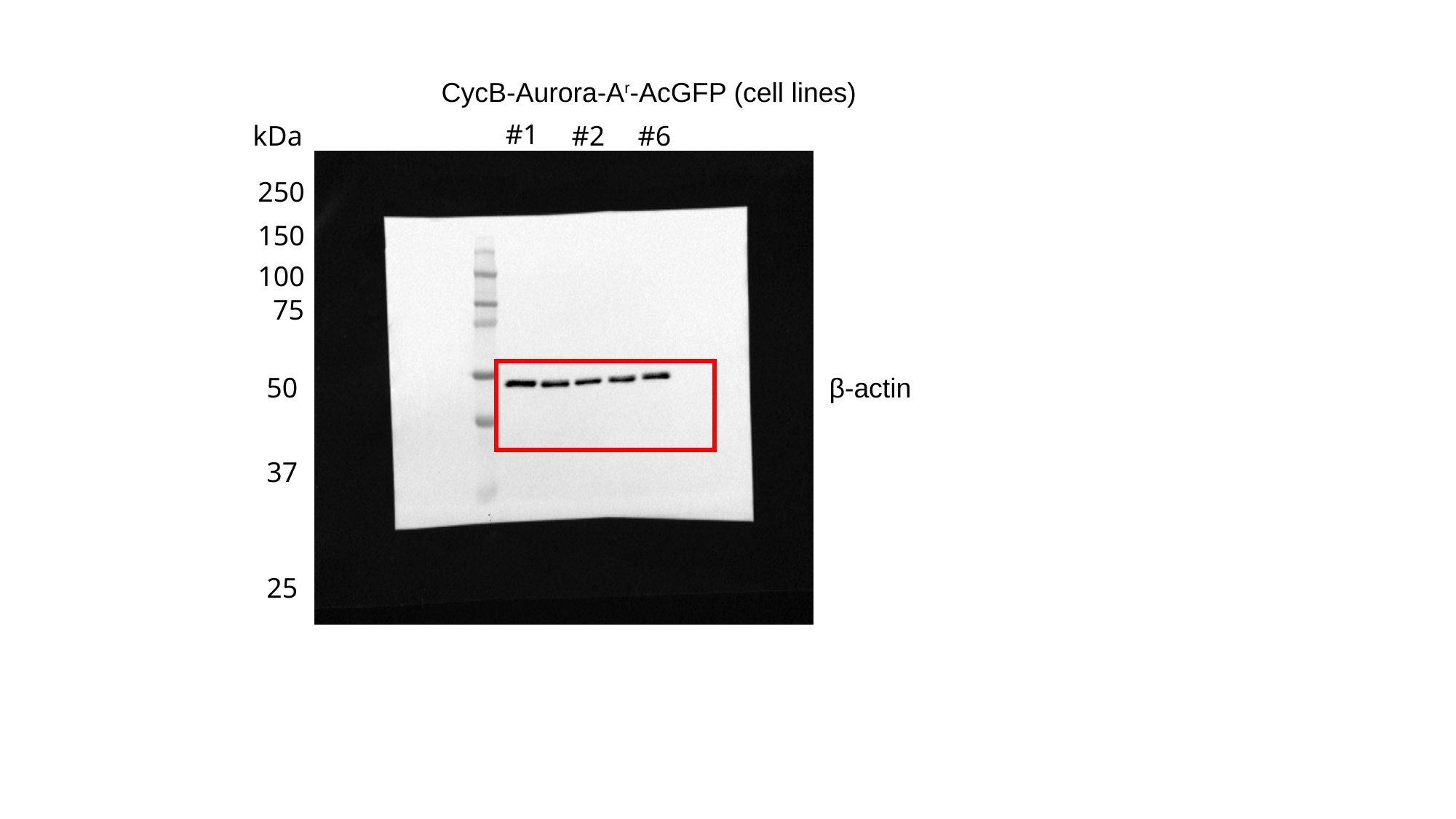

CycB-Aurora-Ar-AcGFP (cell lines)
#1
kDa
#2
#6
250
150
100
75
50
β-actin
37
25
