# Supplementary material for: Aurora A regulates the material property of spindle poles to orchestrate nuclear organization at mitotic exit
Source: EMBO J. 2025 Sep 12;44(23):6797–831. doi: 10.1038/s44318-025-00564-4 (PMC12669695; doi:10.1038/s44318-025-00564-4)
Supplement: Supplementary file 17 — Expanded View Figures [file 44318_2025_564_MOESM17_ESM.pdf]

Expanded View Figures

Figure EV1. Anaphase-specific Aurora A degradation tool to study Aurora A function at mitotic exit.

(**A**) Immunofluorescence (IF) analysis of anaphase cells stained with phospho-specific anti-Aurora A antibody against T288 ($T288^p$; green). DNA is shown in blue. (**B**) Schematic of the method to quantify the $T288^p$ enrichment (in au) at poles in cells stained with anti-$T288^p$ antibody during anaphase. In this and subsequent Fig. panels bkgd., representing background intensity. Note that $T288^p$ is significantly enriched at the poles in anaphase, indicating the presence of active Aurora A pool at the poles during anaphase. Error bars: mean ± SD. (**C**) Immunoblot analysis of protein extracts prepared from mitotically synchronized control HeLa or HeLa cells stably expressing CycB-AcGFP. Extracts were probed with antibodies directed against CyclinB and β-actin. Endogenous CyclinB and CycB-AcGFP bands are indicated. In this and other immunoblot panels, the molecular mass is indicated in kilodaltons (kDa) on the left. (**D**) Schematic of the method and the quantification of the chromosomal intensity of CycB-AcGFP (in au) during the metaphase-to-anaphase transition (time 0). The graph illustrates the mean, and the shaded region indicates ± SEM. For representative images, see 1G. (**E**) IF analysis of control and monoclonal cell line expressing CycB-Aurora A^r-AcGFP. These cells were stained with anti-Aurora A (red; endogenous protein detection) and anti-GFP (green; exogenous protein detection) antibodies upon transfection with control and Aurora A siRNA for 60 h. DNA is shown in blue. (**F**) Quantification of the fraction of mitotic cells (mitotic index) in control cells and monoclonal cell line expressing CycB-Aurora A^r-AcGFP. Cells were analyzed by IF 60 h after transfection with control and Aurora A siRNA. Error bars: mean ± SD from two independent experiments ($n > 1000$ cells each). Exact p values from left to right are $^{**}p = 0.0049$, $p = 0.4392$ (ns). (**G**) Schematic representation of chromosome instability defects and the quantification of such defects in control cells and monoclonal cell line expressing CycB-Aurora A^r-AcGFP upon transfection with control and Aurora A siRNA for 60 h, as indicated. Error bars: mean ± SD from two independent experiments ($n > 120$ cells each). Exact p value is $^{***}p < 0.001$. (**H**) Schematic representation and the estimation of chromosome separation kinetics of monoclonal cell line expressing CycB-Aurora A^r-AcGFP in control or upon transfection with control and Aurora A siRNA for 60 h. Values represent mean ± SD. Exact p values from left to right are $^{***}p < 0.001$, $^{***}p < 0.001$, $^{***}p < 0.001$. (**I**) Confocal live-cell imaging of HeLa cells stably expressing AuroraA^r-AcGFP (green) and probed for silicon-rhodamine DNA (SiR-DNA; magenta) to visualize chromosomes ensemble and nuclear shape post-mitosis in control and upon transfection with Aurora A siRNA for 60 h. Also, see nuclear shape analysis of cells expressing CycB-AuroraA^r-AcGFP in control and upon Aurora A depletion in Fig. 1I–K. (**J**, **K**) Nuclear shape analysis [circularity (**J**) and solidity (**K**)] from the confocal live-cell imaging of cells, as mentioned in (**I**). Exact p values are $p = 0.4962$ (ns) (**J**) and $p = 0.0813$ (ns) (**K**). p values are denoted as follows: ns-$p \geq 0.05$; $^{**}p < 0.01$; $^{***}p < 0.001$ as determined by two-tailed unpaired Student's t-test. Scale bars in (**A**, **E**, **I**) represent 10 μm.

© The Author(s)

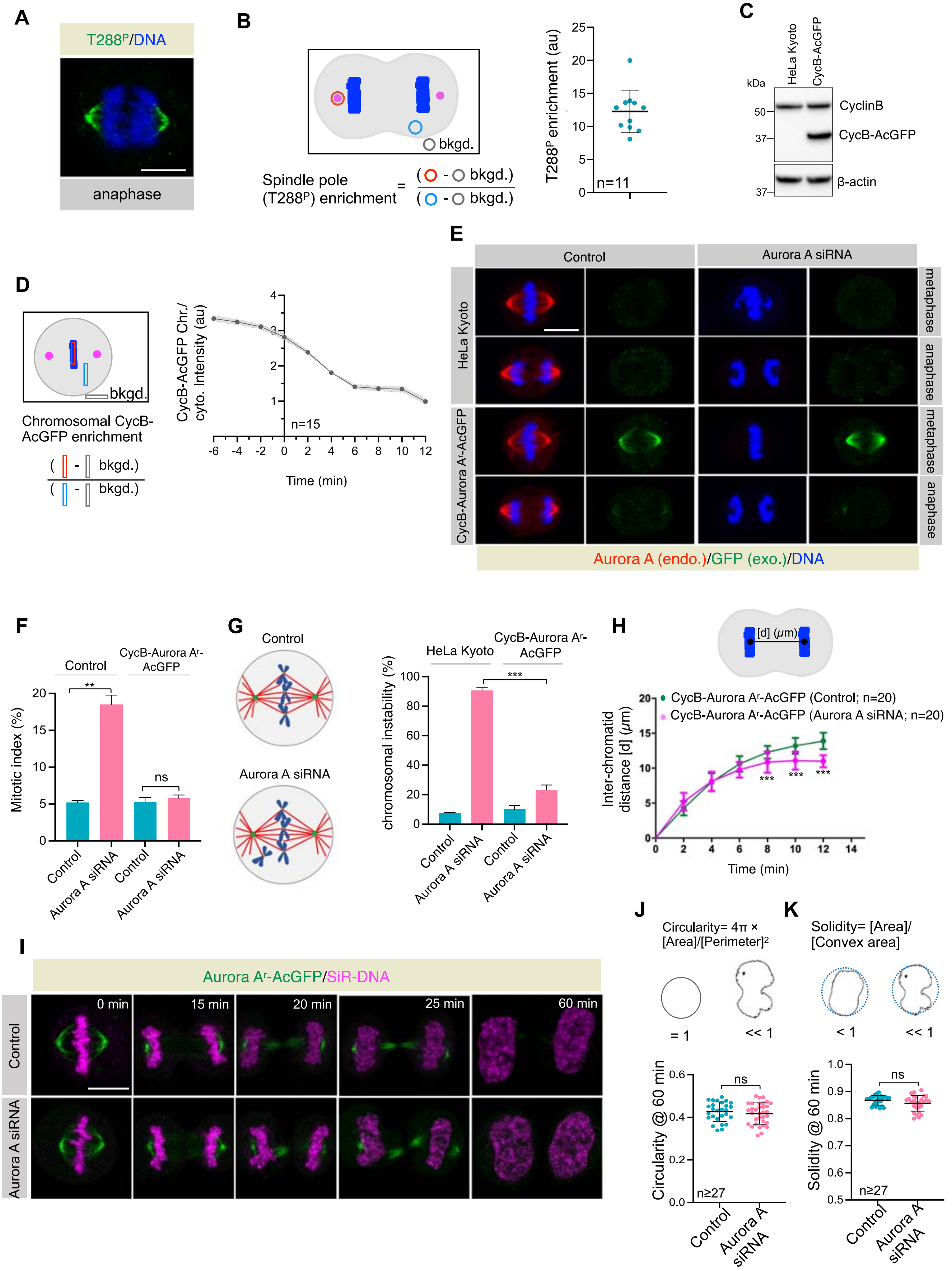
A
T288P/DNA
anaphase
B
bkgd.
Spindle pole (T288P) enrichment
bkgd.
T288P enrichment (au)
n=11
C
HeLa Kyoto
CycB-AcGFP
kDa
CyclinB
CycB-AcGFP
β-actin
D
bkgd.
Chromosomal CycB-AcGFP enrichment
CycB-AcGFP Chr./ cyto. Intensity (au)
n=15
Time (min)
E
Control
Aurora A siRNA
HeLa Kyoto
CycB-Aurora Ar-AcGFP
metaphase
anaphase
Aurora A (endo.)/GFP (exo.)/DNA
F
Control
CycB-Aurora Ar-AcGFP
Mitotic index (%)
ns
Aurora A siRNA
G
Control
Aurora A siRNA
HeLa Kyoto
chromosomal instability (%)
H
[d] (μm)
CycB-Aurora Ar-AcGFP (Control; n=20)
CycB-Aurora Ar-AcGFP (Aurora A siRNA; n=20)
Inter-chromatid distance [d] (μm)
Time (min)
I
Aurora Ar-AcGFP/SiR-DNA
0 min
15 min
20 min
25 min
60 min
J
Circularity= 4π × [Area]/[Perimeter]2
= 1
<< 1
Circularity @ 60 min
n≥27
K
Solidity= [Area]/ [Convex area]
< 1
<< 1
Solidity @ 60 min
n≥27

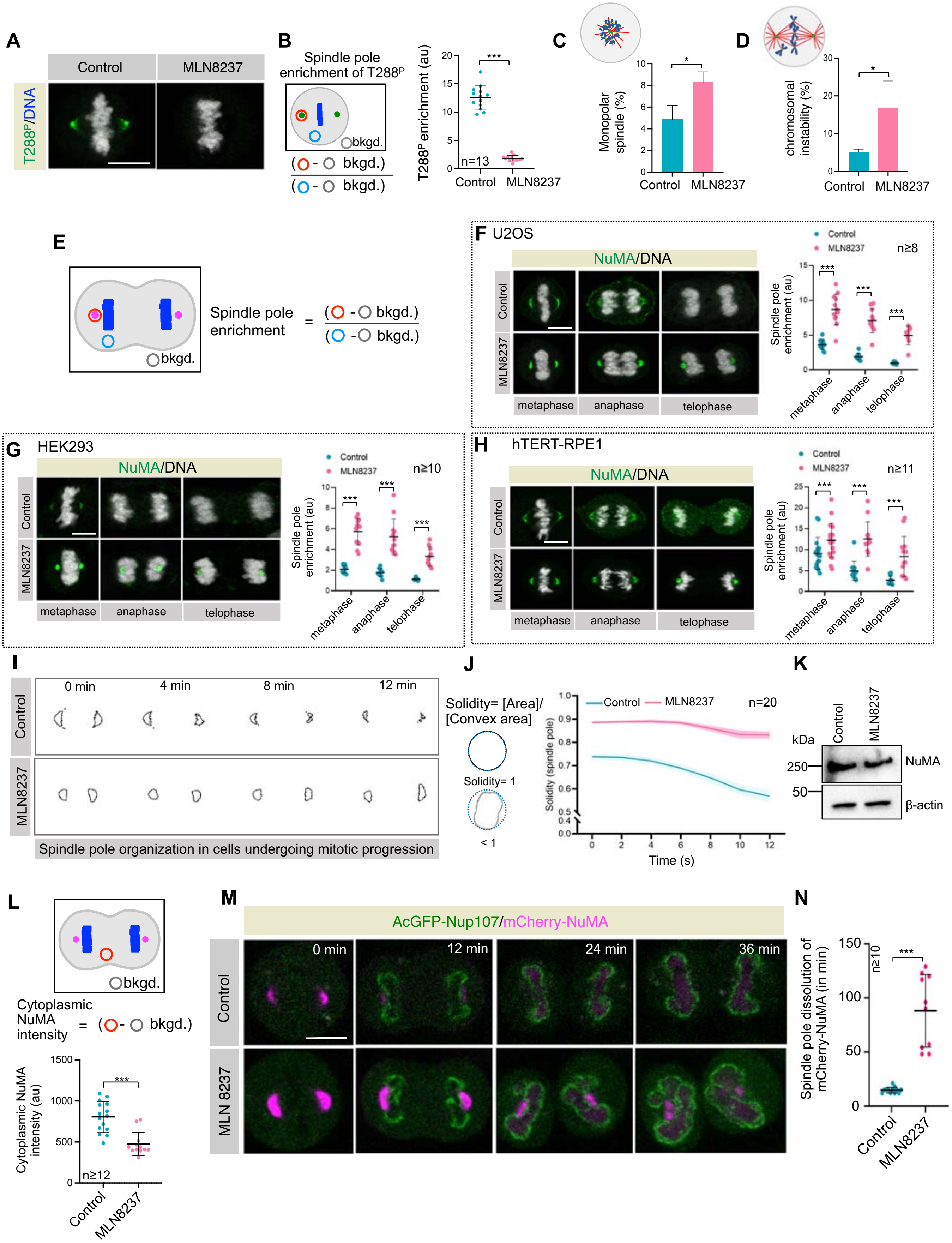
A
Control
MLN8237
T288P/DNA
B
Spindle pole enrichment of T288P
bkgd.
(- bkgd.)
(- bkgd.)
T288P enrichment (au)
n=13
Control
MLN8237
C
Monopolar spindle (%)
Control
MLN8237
D
chromosomal instability (%)
Control
MLN8237
E
bkgd.
Spindle pole enrichment = (- bkgd.) / (- bkgd.)
F U2OS
NuMA/DNA
Control
MLN8237
metaphase
anaphase
telophase
n≥8
Spindle pole enrichment (au)
G HEK293
NuMA/DNA
Control
MLN8237
metaphase
anaphase
telophase
n≥10
Spindle pole enrichment (au)
H hTERT-RPE1
NuMA/DNA
Control
MLN8237
metaphase
anaphase
telophase
n≥11
Spindle pole enrichment (au)
I
0 min
4 min
8 min
12 min
Control
MLN8237
Spindle pole organization in cells undergoing mitotic progression
J
Solidity= [Area]/ [Convex area]
Solidity= 1
< 1
Control
MLN8237
n=20
Solidity (spindle pole)
Time (s)
K
kDa
Control
MLN8237
250
50
NuMA
β-actin
L
bkgd.
Cytoplasmic NuMA intensity = (- bkgd.)
Cytoplasmic NuMA intensity (au)
n≥12
Control
MLN8237
M
AcGFP-Nup107/mCherry-NuMA
0 min
12 min
24 min
36 min
Control
MLN 8237
N
Spindle pole dissolution of mCherry-NuMA (in min)
n≥10
Control
MLN8237

 © The Author(s)

Figure EV2. NuMA is abnormally accumulated at the poles upon acute Aurora A inhibition at the mitotic exit.

(**A**) IF analysis of HeLa cells during metaphase in control and upon acute Aurora A inhibition (2 h) with MLN8237 (50 nM). Cells were stained for $T288^P$ (green). DNA is shown in gray. (**B**) Schematic representation of the quantification method and the quantification of $T288^P$ at poles in cells stained with anti-$T288^P$ antibody in metaphase. Note that $T288^P$ enrichment is significantly affected in cells treated with Aurora A inhibitor MLN8237. Error bars: mean ± SD. Exact *p* value is ***$p < 0.001$. (**C**, **D**) Quantification of the fraction of monopolar spindle (**C**), and chromosomal instability defects (**D**) in control and upon acute Aurora A inhibition (2 h) with MLN8237 (50 nM). Error bars: mean ± SD from three independent experiments ($n > 300$ cells each). Note that cells without any sign of chromosome instability were utilized for further analysis. Exact *p* values are *$p = 0.0113$ (**C**) and *$p = 0.0266$ (**D**). (**E**–**H**) Schematic of the method to quantify spindle pole enrichment of NuMA (in au) (**E**) and the outcome of such analysis by performing IF in various cell lines such as U2OS (**F**), HEK293 (**G**), and hTERT-RPE1 (**H**) in control and upon acute Aurora A inhibition with MLN8237 (50 nM) for 2 h. For this analysis, cells were stained with anti-NuMA (green) antibody. DNA is shown in gray. The quantification on the right represents mean ± SD. Exact *p* values from left to right in (**F**–**H**) are ***$p < 0.001$, ***$p < 0.001$, ***$p < 0.001$. (**I**, **J**) The representative geometry of NuMA organization at the pole in control and MLN8237-treated HeLa cells. Timepoint $t = 0$ min was set to the metaphase-to-anaphase transition (**I**). The measurement of the solidity of NuMA-based poles in control and MLN8237-treated cells is shown in (**J**). Curves, and shaded areas indicate mean ± SEM. The p-values for the solidity of NuMA-based poles for all the time points is <0.001. (**K**) Immunoblot analysis of protein extracts prepared from anaphase-synchronized control and MLN8237-treated cells. Extracts were probed with anti-NuMA and anti-β-actin antibodies. (**L**) Schematic of the method to quantify cytoplasmic NuMA intensity during anaphase and the outcome of such analysis (in au). Bars indicate mean ± SD. Exact *p* value is ***$p < 0.001$. (**M**, **N**) Confocal live-cell imaging of HeLa cells stably expressing nucleoporin marker AcGFP-Nup107 (green) and were transiently transfected with mCherry-NuMA (magenta) in the absence (control) or upon acute inhibition of Aurora A kinase using MLN8237 (**M**). Timepoint $t = 0$ min was set to the metaphase-to-anaphase transition. Quantification of the dissolution time (in min) of mCherry-NuMA at the poles in control and MLN8237-treated cells with respect to (w.r.t.) metaphase-to-anaphase transition (**N**). Error bars: mean ± SD. Exact *p* value is ***$p < 0.001$. *p* values are denoted as follows: *$p < 0.05$; ***$p < 0.001$ as determined by two-tailed unpaired Student's *t*-test. Scale bars in (**A**, **F**, **G**, **H**, **M**) represent 10 µm.

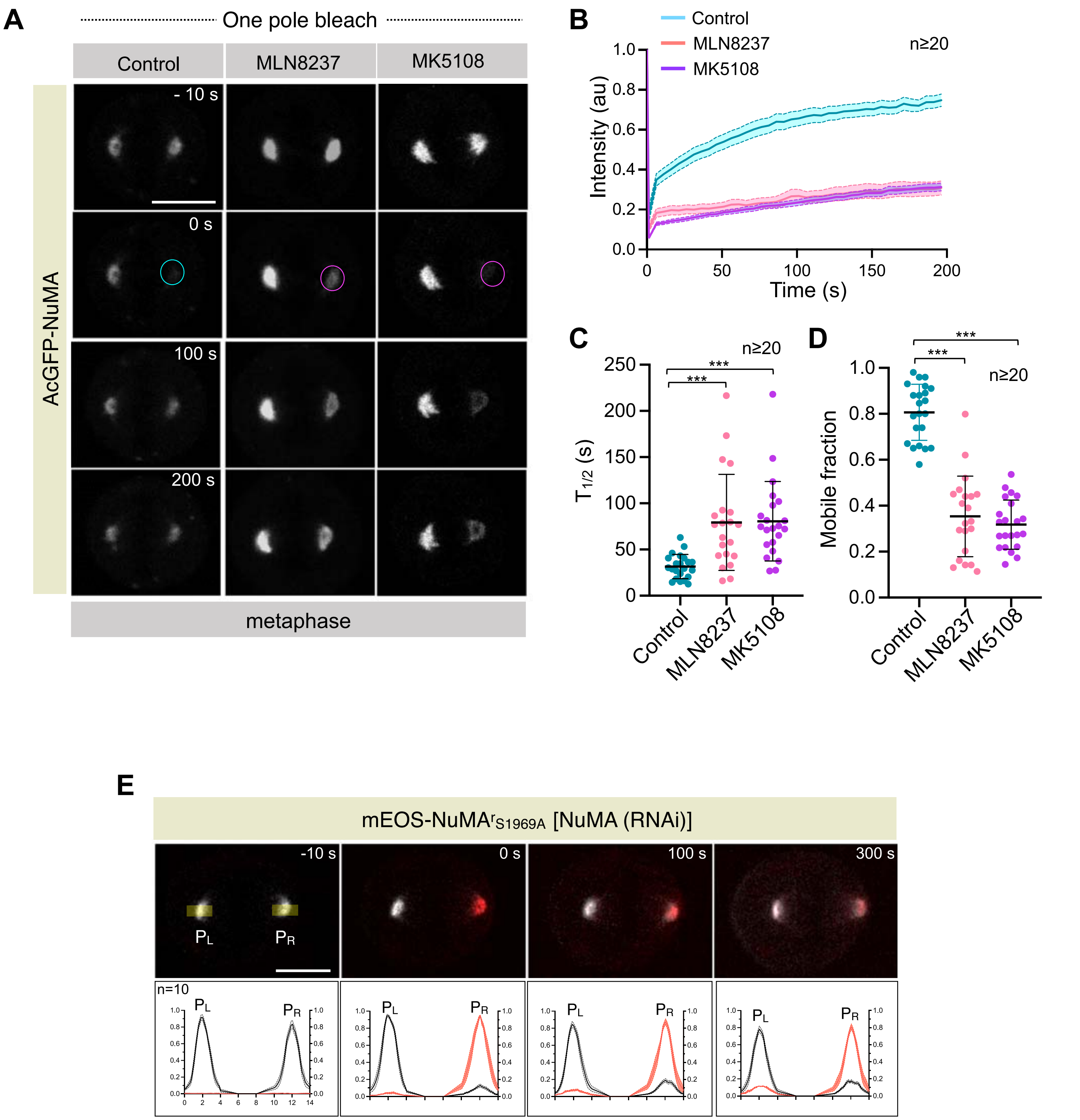
A
One pole bleach
Control
MLN8237
MK5108
AcGFP-NuMA
- 10 s
0 s
100 s
200 s
metaphase
B
Control
MLN8237
MK5108
n≥20
Intensity (au)
1.0
0.8
0.6
0.4
0.2
0.0
0
50
100
150
200
Time (s)
C
n≥20
***
***
T1/2 (s)
250
200
150
100
50
0
Control
MLN8237
MK5108
D
***
***
n≥20
Mobile fraction
1.0
0.8
0.6
0.4
0.2
0.0
Control
MLN8237
MK5108
E
mEOS-NuMAr S1969A [NuMA (RNAi)]
-10 s
0 s
100 s
300 s
PL
PR
n=10

 © The Author(s)

Figure EV3. NuMA changes its material properties from dynamic-to-solid upon Aurora A inhibition.

(**A**) FRAP analysis of metaphase cells stably expressing AcGFP-NuMA (gray) that are either left untreated or treated with specific Aurora A inhibitors – MLN8237 or MK5108, as indicated. Time is indicated in seconds (s). Blue and magenta circles show the bleached regions of control and MLN8237 or MK5108-treated cells, respectively. (**B**) The AcGFP recovery profile of the bleached area is plotted for 200 s for control and MLN8237 or MK5108-treated cells. Curves and shaded areas indicate mean ± SEM. Note the remarkably slow recovery of pole-localized AcGFP-NuMA signal in MLN8237 or MK5108-treated cells. (**C**, **D**) The half-time of recovery [$T_{1/2}$] (**C**) and the mobile fraction (**D**) of untreated and MLN8237 or MK5108-treated metaphase cells. Error bars: mean ± SD. See Methods for details. Exact *p* values from left to right are ***$p < 0.001$, ***$p < 0.001$ for (**C**) and ***$p < 0.001$, ***$p < 0.001$ for (**D**). See Methods for details. (**E**) One-pole photoconversion and the dynamics of photoconverted mEOS-NuMA$^{r}_{(S1969A)}$ signal in HeLa cells depleted for endogenous NuMA after 72 h of transfection with NuMA siRNA. Timepoint "0" indicates the time when mEOS-NuMA$^{r}_{S1969A}$ was photoconverted at one pole. A line-scan with a line thickness of 3 µm (roughly similar to the size of the poles) was performed for the unperturbed left pole (P_L) or perturbed right pole (P_R). Ten cells were analyzed (see Methods). Error bars: mean ± SEM. *p* values are denoted as ***$p < 0.001$ as determined by two-tailed unpaired Student's *t*-test. Scale bars in (**A**, **E**) represent 10 µm.

© The Author(s)

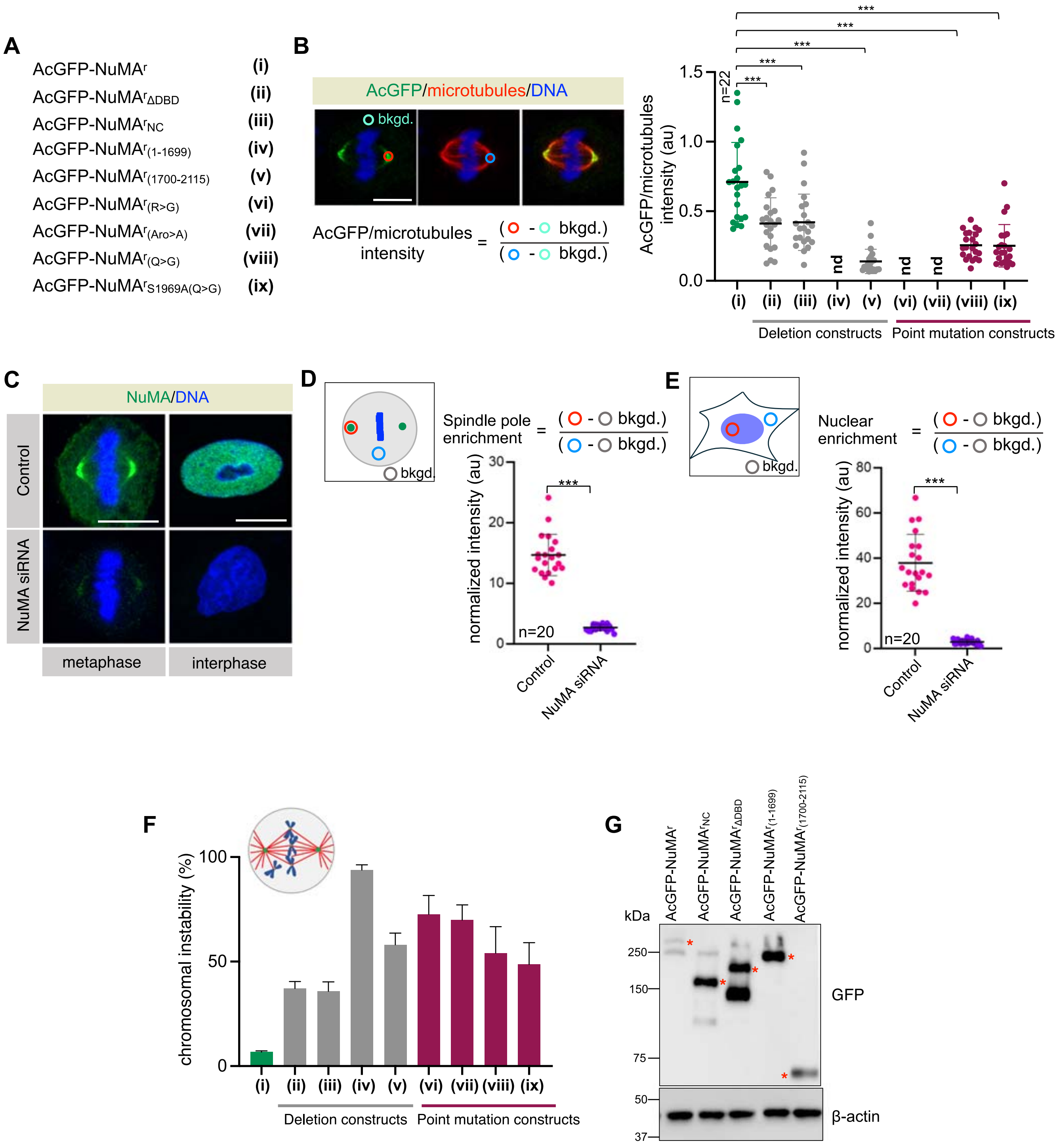

A
AcGFP-NuMAr (i)
AcGFP-NuMArΔDBD (ii)
AcGFP-NuMArNC (iii)
AcGFP-NuMAr(1-1699) (iv)
AcGFP-NuMAr(1700-2115) (v)
AcGFP-NuMAr(R>G) (vi)
AcGFP-NuMAr(Aro>A) (vii)
AcGFP-NuMAr(Q>G) (viii)
AcGFP-NuMArS1969A(Q>G) (ix)
B
AcGFP/microtubules/DNA
bkgd.
AcGFP/microtubules intensity = (- bkgd.) / (- bkgd.)
AcGFP/microtubules intensity (au)
n=22
***
nd
(i) (ii) (iii) (iv) (v) (vi) (vii) (viii) (ix)
Deletion constructs
Point mutation constructs
C
NuMA/DNA
Control
NuMA siRNA
metaphase
interphase
D
bkgd.
Spindle pole enrichment = (- bkgd.) / (- bkgd.)
normalized intensity (au)
n=20
Control
NuMA siRNA
E
bkgd.
Nuclear enrichment = (- bkgd.) / (- bkgd.)
normalized intensity (au)
n=20
Control
NuMA siRNA
F
chromosomal instability (%)
(i) (ii) (iii) (iv) (v) (vi) (vii) (viii) (ix)
Deletion constructs
Point mutation constructs
G
AcGFP-NuMAr
AcGFP-NuMArNC
AcGFP-NuMArΔDBD
AcGFP-NuMAr(1-1699)
AcGFP-NuMAr(1700-2115)
kDa
250
150
75
50
37
GFP
β-actin

 © The Author(s)

Figure EV4. Expression of various NuMA mutant or deletion constructs lead to significant chromosomal instability.

(**A**) Nomenclature of siRNA-resistant wild-type full-length NuMA (**i**) and various deletion and mutant constructs (**ii–ix**), as indicated. See Figs. 4, 5, for detail. (**B**) Schematic representation of the method, and the quantification of spindle pole intensity in terms of AcGFP/microtubules intensity (in au) in HeLa cells expressing either wild-type (**i**) or deletion/mutant NuMA constructs (**ii–ix**), as mentioned in (**A**), 72 h after transfections with NuMA siRNA. These cells were stained with anti-GFP (green), and anti-α-tubulin (red) antibodies. The DNA is shown in blue. Please note, that (**iv**), (**vi**), and (**vii**) fail to localize at the poles, and therefore their ratio with the microtubules cannot be determined (nd). Exact p values from left to right are ***$p < 0.001$, ***$p < 0.001$, ***$p < 0.001$, ***$p < 0.001$, ***$p < 0.001$. (**C–E**) IF analysis to assess the efficiency of NuMA depletion after 72 h of transfection with NuMA siRNA. IF analysis shows NuMA localization during mitosis and in interphase (**C**) and NuMA quantification at the poles (**D**) and in the nucleus (**E**). NuMA was stained using anti-NuMA antibodies (green), and DNA is shown in blue. Error bars: mean ± SD. Exact p values are ***$p < 0.001$ (**D**) and ***$p < 0.001$ (**E**). (**F**) Quantification of the fraction of chromosomal instability in HeLa cells expressing either wild-type (**i**) or deletion/mutant NuMA constructs (**ii–ix**; see **A**), 72 h after transfections with NuMA siRNA. Error bars represent mean ± SD of two experiments ($n > 100$ cells each). Note a direct correlation between failure in pole localization by these mutants and an increase in chromosomal instability. Also note that the ability of these cells to organize the quasi-bipolar spindle could be due to the residual endogenous NuMA levels in our experimental conditions. (**G**) Immunoblot analysis of protein extracts prepared from prometaphase synchronized cells expressing either wild-type or various deletion constructs of NuMA tagged with AcGFP, as indicated. Extracts were probed with anti-GFP and anti-β-actin antibodies. Asterisk represents the correct size of the AcGFP-tagged protein. p values are denoted as ***$p < 0.001$ as determined by two-tailed unpaired Student's *t*-test. Scale bars in (**B**, **C**) represent 10 µm.

© The Author(s)

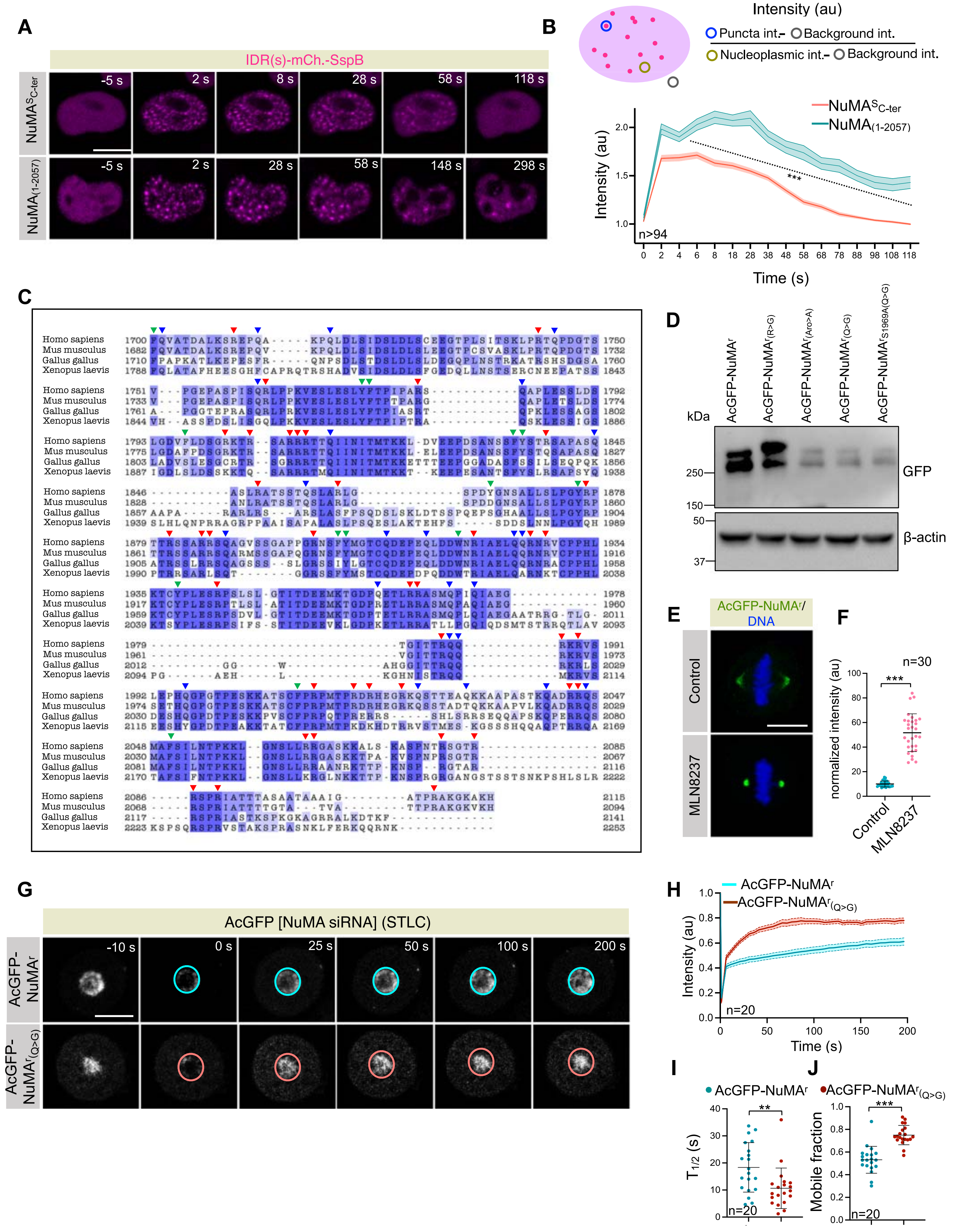
A
IDR(s)-mCh.-SspB
NuMA$^{S}_{C\text{-ter}}$
-5 s
2 s
8 s
28 s
58 s
118 s
NuMA(1-2057)
-5 s
2 s
28 s
58 s
148 s
298 s
B
Intensity (au)
Puncta int.- Background int.
Nucleoplasmic int.- Background int.
NuMA$^{S}_{C\text{-ter}}$
NuMA(1-2057)
Intensity (au)
***
n>94
Time (s)
C
Homo sapiens
Mus musculus
Gallus gallus
Xenopus laevis
D
kDa
AcGFP-NuMAʳ
AcGFP-NuMAʳ(R>G)
AcGFP-NuMAʳ(Aro>A)
AcGFP-NuMAʳ(Q>G)
AcGFP-NuMAʳS1969A(Q>G)
250
150
50
37
GFP
β-actin
E
AcGFP-NuMAʳ/ DNA
Control
MLN8237
F
n=30
***
normalized intensity (au)
Control
MLN8237
G
AcGFP [NuMA siRNA] (STLC)
AcGFP-NuMAʳ
AcGFP-NuMAʳ(Q>G)
-10 s
0 s
25 s
50 s
100 s
200 s
H
AcGFP-NuMAʳ
AcGFP-NuMAʳ(Q>G)
Intensity (au)
n=20
Time (s)
I
AcGFP-NuMAʳ
T1/2 (s)
**
n=20
J
AcGFP-NuMAʳ(Q>G)
Mobile fraction
***
n=20

 © The Author(s)

Figure EV5. Glutamine residues in the NuMA C-terminus provide hardening to the poles.

(**A**) Confocal live-cell imaging of Corelet-expressing HEK293 cells with wild-type $NuMA^{S}_{C\text{-}ter}$ or full-length NuMA lacking its chromatin binding domain [$NuMA_{(1–2057)}$]. Note that the condensates of $NuMA_{(1–2057)}$ are significantly slower in dissolving compared to $NuMA^{S}_{C\text{-}ter}$ IDR. Please note the re-use of the image (Fig. 5C) revealing $NuMA^{S}_{C\text{-}ter}$ expression. (**B**) Schematic representation of the quantification method, intensity (in au), and the dynamics of Corelet-based condensates —$NuMA^{S}_{C\text{-}ter}$, or $NuMA_{(1–2057)}$. Curves and shaded areas indicate mean ± SEM. As indicated the behavior of more than 94 clusters were analysed from a minimum of 20 cells in each condition. (**C**) Sequence alignment of the NuMA C-terminus (1700–2115 aa) of different NuMA orthologs. Red, green, and blue arrowheads represent arginine (R), aromatic (Aro: W, Y, F), and glutamine (Q) residues, respectively. (**D**) Immunoblot analysis of protein extracts prepared from prometaphase synchronized cells expressing either wild-type or various AcGFP-tagged NuMA mutants, as indicated. Extracts were probed with anti-GFP and anti-β-actin antibodies. Since NuMA is highly phosphorylated in mitosis, the two bands detected by anti-GFP antibodies could be phosphorylated and non-phosphorylated NuMA bands. (**E**, **F**) Representative images of HeLa cells stably expressed low levels of siRNA resistant AcGFP-tagged NuMA (in green) that were depleted for endogenous NuMA by siRNA. These cells are either treated with DMSO (control) or treated with MLN8237, as indicated (**E**). DNA is shown in blue. Normalized spindle pole intensity (in au) on the right of AcGFP-$NuMA^{r}$ at the poles in control and MLN8237-treated cells (**F**). Error bars ± SD. Exact p value is $^{***}p < 0.001$. (**G**) FRAP analysis of HeLa cells which are either expressing AcGFP-$NuMA^{r}$ (gray) or AcGFP-$NuMA^{r}_{(Q>G)}$ upon endogenous protein depletion for 72 h. These cells were treated with *S*-trityl-L-cysteine (STLC)—kinesin 5 inhibitor for 17 h before imaging to block bipolar spindle assembly. Time is indicated in seconds (s). Blue and orange circles show the bleached regions of AcGFP-$NuMA^{r}$ and AcGFP-$NuMA^{r}_{(Q>G)}$ expressing cells, respectively. Note the significantly faster recovery of AcGFP signal in cells expressing AcGFP-$NuMA^{r}_{(Q>G)}$. (**H**) The AcGFP recovery profile of the bleached area corrected for photobleaching is plotted for 200 s for AcGFP-$NuMA^{r}$ and AcGFP-$NuMA^{r}_{(Q>G)}$ expressing cells. Curves and shaded areas indicate mean ± SEM. (**I**, **J**) The half-time of recovery [$T_{1/2}$] in (s) (**I**) and the mobile fraction (**J**) of AcGFP-$NuMA^{r}$ and AcGFP-$NuMA^{r}_{(Q>G)}$ expressing cells, as indicated. Error bars: mean ± SD. Exact p values are $^{**}p < 0.0044$ (**I**) and $^{***}p < 0.001$ (**J**). p values are denoted as follows: $^{**}p < 0.01$; $^{***}p < 0.001$ as determined by two-tailed unpaired Student's *t*-test. Scale bars in (**A**, **E**, **G**) represent 10 µm.

© The Author(s)
